# Trans* people’s access to gender-affirming health care: A European comparison

**DOI:** 10.1093/eurpub/ckac129.070

**Published:** 2022-10-25

**Authors:** J Breckenkamp, J Thirugnanamohan, A Stern, O Razum, Y Namer

**Affiliations:** 1 School of Public Health, Bielefeld University, Bielefeld, Germany; 2 Research Institute Social Cohesion, Bielefeld, Germany

## Abstract

**Background:**

Trans* people's life satisfaction is correlated with established legal frameworks for gender recognition and access to trans*-specific health care (Transgender Europe [TGEU], 2021). TGEU's guidelines to human rights-based principles of trans*-specific health care highlight bodily integrity/autonomy, free self-determination of gender, quality, specialized and decentralized care, and the right to determine reproductive paths as important pillars of gender affirming health care. We conducted a policy comparison across Europe regarding access to gender-affirming health care to assess how adherence to human rights-based principles could be strengthened.

**Methods:**

We compared access to health care across four main domains: legal framework (e.g., legally recognised genders), insurance coverage (e.g., out of pocket costs), access barriers (e.g., legal requirements to access gender-affirming surgery), and health care offers (e.g., hormone replacement therapy). Criteria were developed in guided brainstorming sessions. Three researchers rated 28 countries across 28 items based on available policy documents.

**Results:**

The majority of European countries prescribes a medicalised gender-affirming process rather than a self-decided process. Psychiatric diagnosis is also required in most countries to access gender-affirming health care. Gender-affirming health care is partly financed by statutory health insurance in most of the countries. Not all countries authorise full gender-affirming health care. Especially where statutory health insurance-covered gender-affirming health care relied centralised on single outpatient clinics or hospitals, waiting times between 6-24 months are found.

**Conclusions:**

Many European countries fail to fully comply with TGEU's guidelines to human rights-based principles of trans* health care. Given the negative impact of access barriers on life satisfaction, European countries should target these shortfalls in ensuring gender-affirming health care.

**Key messages:**

• Non- or only partly covered trans* health care contributes to health inequality.

• Regarding trans* people, European countries need to strengthen human-rights based access to gender-affirming health care.

